# The influence of tumour vasculature on fluid flow in solid tumours: a mathematical modelling study

**DOI:** 10.52601/bpr.2021.200041

**Published:** 2021-02-28

**Authors:** Moath Alamer, Xiao Yun Xu

**Affiliations:** 1 Department of Chemical Engineering Imperial College London, South Kensington Campus, London, United Kingdom

**Keywords:** Fluid flow, Vasculature, Tumour, Mathematical modelling

## Abstract

Tumour vasculature is known to be aberrant, tortuous and erratic which can have significant implications for fluid flow. Fluid dynamics in tumour tissue plays an important part in tumour growth, metastasis and the delivery of therapeutics. Mathematical models are increasingly employed to elucidate the complex interplay between various aspects of the tumour vasculature and fluid flow. Previous models usually assume a uniformly distributed vasculature without explicitly describing its architecture or incorporate the distribution of vasculature without accounting for real geometric features of the network. In this study, an integrated computational model is developed by resolving fluid flow at the single capillary level across the whole tumour vascular network. It consists of an angiogenesis model and a fluid flow model which resolves flow as a function of the explicit vasculature by coupling intravascular flow and interstitial flow in tumour tissue. The integrated model has been used to examine the influence of microvascular distribution, necrosis and vessel pruning on fluid flow, as well as the effect of heterogeneous vessel permeability. Our results reveal the level of nonuniformity in tumour interstitial fluid pressure (IFP), with large variations in IFP profile between necrotic and non-necrotic tumours. Changes in microscopic features of the vascular network can significantly influence fluid flow in the tumour where removal of vessel blind ends has been found to reduce IFP and promote interstitial fluid flow. Our results demonstrate the importance of incorporating microscopic properties of the tumour vasculature and intravascular flow when predicting fluid flow in tumour tissue.

## INTRODUCTION

Fluid flow in tissue plays an important role in the delivery of oxygen, nutrients and therapeutic agents. The physiology of tumour tissues exhibits several distinct features that influence fluid flow which can potentially promote tumour invasiveness and limit drug delivery. As tumours grow beyond the limit at which preexisting vasculature is able to sufficiently deliver blood, hypoxia develops which can trigger angiogenesis (Carmeliet and Jain 2000; Liao and Johnson 2007). This leads to the formation of aberrant vascular networks with non-uniformly distributed blood vessels that lack hierarchy and differentiation. Excessive branching can be found with loops, blind ends, arteriovenous shunts and erratic changes in diameter which can result in impaired blood perfusion (Less *et al*. 1991). Cellular abnormalities of blood vessels lead to poor vessel stability and large inter-endothelial gaps that allow for excessive leakage of plasma (Baluk *et al*. 2005). In the tumour extravascular space, the extracellular matrix (ECM) is produced at a high rate, generating mechanical stress which combined with the leaky vessels leads to high interstitial fluid pressure (IFP) (Heldin *et al*. 2004). These properties act together to cause abnormal fluid flow in tumours which can be heterogenous and vary from patient to patient (Swartz and Lund 2012). Most therapeutics are injected intravenously and need to reach the tumour site, distribute within the vascular network, permeate through the vessel wall and travel across the interstitial space to reach cancer cells (Zhan *et al*. 2018). Hence, abnormal blood flow, reduced extravasation and pressure gradients caused by high IFP can result in poor accumulation and distribution of therapeutic macromolecules. Additionally, high IFP and poor perfusion have been associated with increased metastatic potential and poor prognosis (Hompland *et al*. 2014; Munson and Shieh 2014). Therefore, gaining deep insight into fluid dynamics in tumour tissue is necessary as it plays a key role in tumour growth, metastasis and the delivery of therapeutics (Dewhirst and Secomb 2017).

Experimental methods have been applied to examine fluid flow in tumour tissues, however, they are limited by the scale and resolution at which they are able to resolve fluid dynamics (Boucher *et al*. 1990; Gulliksrud *et al*. 2009). Mathematical modelling offers a cost-effective approach to gaining quantitative understanding of fluid flow in solid tumours at multiple scales. Several mathematical models have been developed to simulate flow in solid tumours (*e.g*., Baxter and Jain 1989; Soltani and Chen 2011, 2012; Zhan *et al*. 2017). These models treated the tumour vasculature as a uniformly distributed source term without accounting for spatial heterogeneities in vascular distribution. However, tumour vasculature is known to be heterogenous which can lead to heterogenous distribution of molecules and liposomes in tumour tissue (Bhandari *et al*. 2017a; Vavra *et al*. 2004). The influence of tumour vascular heterogeneity on interstitial and transvascular flow has been investigated by several authors (Mohammadi and Chen 2015; Soltani and Chen 2013; Welter and Rieger 2013; Wu *et al*. 2008). In some of these studies, macroscopic parameters for the whole tumour, such as the surface vascular density term, were predefined based on data in the literature rather than considering the morphology and architecture of the network generated. Other studies applied homogenization methods where properties of the vasculature were averaged over discretized cells. By applying Starlings law using these homogenization techniques, only an approximation of the effect of vascular distribution on fluid flow could be investigated. Furthermore, these models treated intravascular flow and interstitial flow in a decoupled manner, so that the effect of transvascular leakage on blood flow was not captured. Although several recent studies addressed the effect of heterogeneous vasculature by making use of contrast-enhanced magnetic resonance imaging (Bhandari *et al*. 2017b, 2018; Zhan *et al*. 2014), these models did not explicitly describe the microvasculature and intravascular flow. The high permeability of tumour vasculature results in excessive fluid leakage from the vessels, hence a strong coupling between vascular and interstitial flow is needed.

Several studies have addressed the coupling between vascular and interstitial flow at the single capillary level (Baish *et al*. 1997; Netti *et al*. 1996). Pozikridis and Farrow developed a Green’s function based mathematical model where sources and dipoles are distributed along a cylindrical vessel segment describing the distribution of vascular and interstitial pressure over the inner and outer surface of the vessels (Pozrikidis and Farrow 2003). This formulation reduces errors introduced by homogenization methods and allows for the interstitial pressure to be described as a function of the architecture and orientation of the vasculature. The model captures the strong coupling of flows by accounting for the influence of extravasation and IFP on intravascular flow. Pozrikidis and Farrow demonstrated that while neglecting the influence of IFP on intravascular flow might be acceptable when vascular hydraulic conductivity is low as in normal tissue, it could result in significant overestimation of transvascular leakage in tumour tissue where vascular hydraulic conductivity is high. So far, Pozrikidis’s fluid flow model has been applied to a single capillary level or a network of ordered vessels (Pozrikidis 2010). Recently, a similar Green’s function based fluid flow model was applied to a vascular network obtained from optical images of cleared tumour tissues (Sweeney *et al*. 2019), where blood flow was assumed to be conserved at each junction and was not influenced by interstitial flow. In all the models reviewed so far, the permeability of the vasculature was assumed to be uniform across the vascular network which failed to capture the heterogenous pore size distribution in tumour vasculature as it can range from 7–1200 nm (Hobbs *et al*. 1998). In terms of evaluating fluid flow in tumours, there is a need to develop models that can predict flow in an entire tumour with explicit descriptions of individual vessel features, such as radius, length, orientation and permeability, whilst capturing the strong coupling between intravascular and interstitial flow.

In this work a mathematical model is developed by coupling Anderson and Chaplain’s tumour induced angiogenesis model (Anderson and Chaplain 1998) with Pozrikidis’s fluid flow model (Pozrikidis and Farrow 2003). The angiogenesis model is extended to 3D to accommodate unique features of the tumour vascular network including excessive branching, looping and high tortuosity. The model describes the response of sprouting vessel tips to tumour angiogenic factors (TAF) secreted by tumours cells. TAF distribution is initialised to describe different tumour geometries with varying degrees of necrosis centered in the domain surrounded by normal tissue. A discrete method is used to track the movement of tip endothelial cells in response to random motion, TAF gradients and fibronectin distribution in the tissue. This forms a network which is regulated and discretized where vessels are divided into short cylindrical segments with a specific length and radius. A fluid flow model is then coupled to the vascular network where vascular, transvascular and interstitial flow are described by Poiseuille’s, Starlings and Darcy’s law respectively. These equations are solved using Pozrikidis’s method to capture the strong coupling between vascular and interstitial flow. Applying Pozrikidis’s model, the interstitial pressure is integrated over the vessel surface, hence geometric features of the vessel, including length, radius and orientation are considered when calculating the IFP profile in the tumour. We have extended the model from a single capillary scale to a complex large scale vascular network, and applied the model to different tumour cases to understand the effects of various properties such as vascular architecture, distribution, tumour necrosis and microscopic details of the tumour network. Furthermore, we have modified the model to allow for variations in vascular permeability as a function of vessel maturity and flow conditions, pushing the model a step closer towards capturing the complex characteristics of tumour vasculature.

## RESULTS

### Tumour vascular network

The vascular networks generated using the angiogenesis model were analysed and their morphological parameters were compared with the corresponding properties found in real tumour vasculature. In this work, three models with a normalized radius of 0.25 were simulated with varying degrees of necrosis: a non-necrotic tumour network, a case with a necrotic core accounting for 3% of the total tumour volume and another case with a 26% necrotic core volume. Table 1 shows the values for the morphological parameters (defined in the Methods section) calculated for the three tumour models. The evaluated morphological parameters are within the range reported in the literature, demonstrating that the generated models are representative of real tumour vascular networks. Comparison of the three models suggests that the average vascular density values are similar; this is because as the degree of central necrosis increases, the tips would move away from the core and create more vessels in the peripheral tumour region.

**Table 1:** Morphological and hemodynamic parameters for different tumour networks with varying degrees of necrosis

Parameter	Unit	Non-necrotic	Necrotic (3%)	Necrotic (26%)	Literature data	Reference
Morphological parameter						
Tumour tissue volume	mm^3^	14.137	14.137	14.137	−	−
Vascular density $ \left({V}_{\mathrm{d}}\right) $	%	0.250	0.245	0.226	0.15−1.25	Kuszyk *et al*. 2001; Nagy *et al*. 2012; Sitohy *et al*. 2012
Length density $ \left({L}_{\mathrm{D}}\right) $	mm/mm^3^	10.675	9.740	9.067	10−72	Kim *et al*. 2012
Surface area to volume ratio (vascular) (*S*/*V*)	mm^2^/mm^3^	216.677	211.224	210.328	122−376	Chugh *et al*. 2009
Maximum extravascular diffusion distance (*R*)	μm	172.683	180.779	187.371	30−250	Konerding *et al*. 2001
Mean vessel diameter	μm	16.074	16.833	16.776	5−225	Hashizume *et al*. 2000; Less *et al*. 1991
Mean vessel length	mm	0.191	0.188	0.189	0.06−0.3	Less *et al*. 1991; Pathak *et al*. 2011
Hemodynamic parameter						
Mean flow	nL/min	2.163	1.704	1.379	−	−
Mean velocity	mm/s	0.233	0.209	0.171	0.1−25	Brizel *et al*. 1993; Kamoun *et al*. 2010
Shear stress	Pa	0.699	0.661	0.543	1−10	Pries *et al*. 2001

After evaluating the vascular networks generated for the different tumour models, the fluid flow model was applied and the calculated hemodynamic parameters were averaged over the entire vascular network for comparison with relevant data in the literature. As shown in Table 1, the mean flow, velocity and shear stress obtained with the fluid flow model are within the reported range, showing the ability of the fluid flow model to capture the intravascular fluid dynamics in tumours.

**Figure 1:**
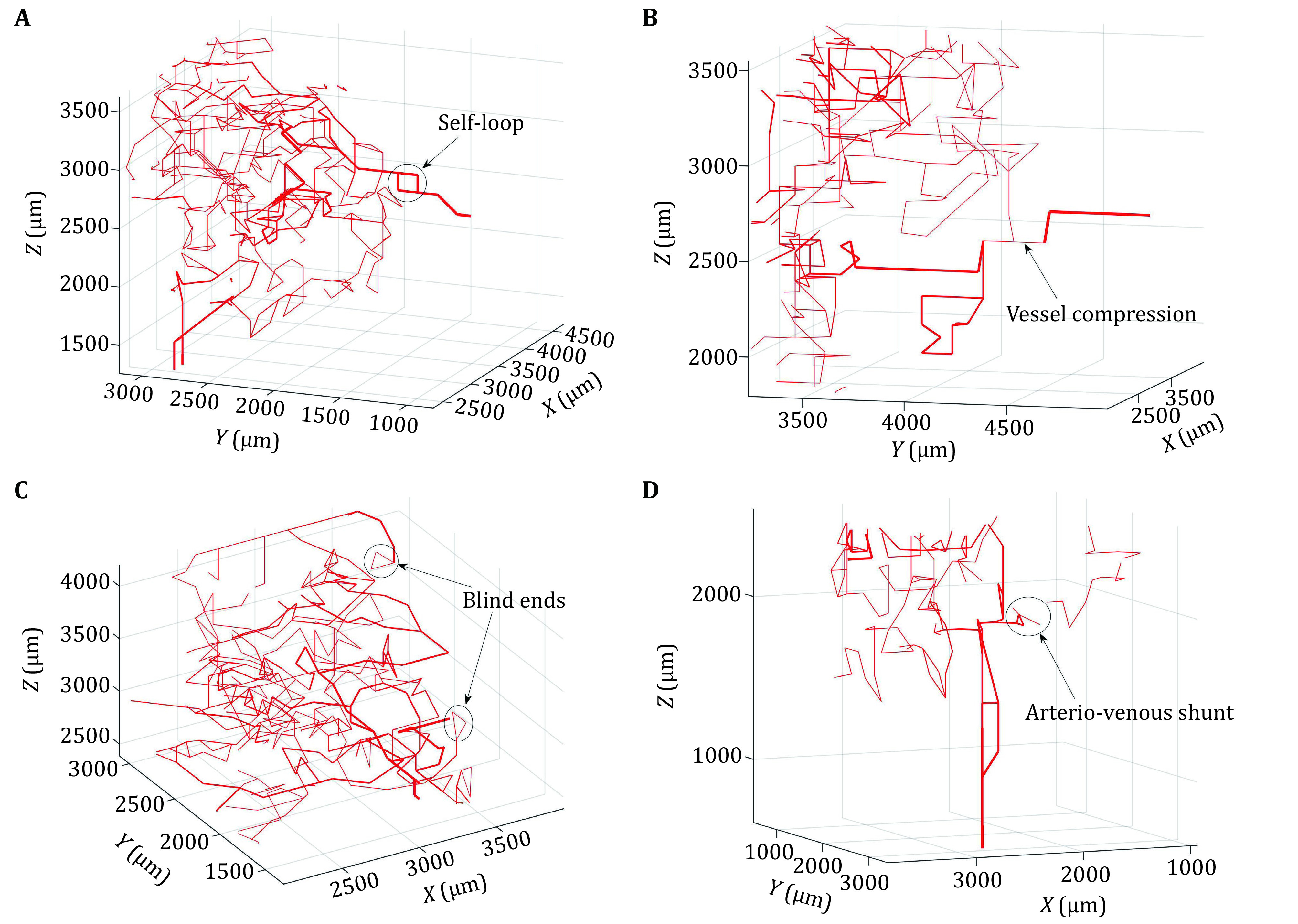
Tumour vascular networks containing abnormal features. **A** Self-loops. **B** Vessel compression. **C** Blind ends. **D** Arteriovenous shunts

It is known that abnormal structural and architectural features, including self-loops, vessel compression and blind ends, are often found in tumour vasculature. The presence of blind ends can cause very low or no flow in some vessels. Another feature is the presence of arteriovenous (AV) shunts where short, low-resistance and high-flow pathways form in close proximity to the feeding vessels. A consequence of these AV shunts is that blood preferentially passes through them due to a large pressure gradient, hence blood can completely bypass the entire capillary network. In the vascular networks described in Table 1, some abnormalities of the tumour vasculature can be captured by modifying the non-necrotic tumour vascular network model (Fig. 1). For example, the default vasculature generated in the non-necrotic model does not contain AV shunts. To simulate this feature, vessels in close proximity to the arterial end are set to branch off where one end is connected to the tumour capillary network and the other end of this branch is assigned a venous pressure boundary condition. This allows for tumour vascular networks with and without AV shunts to be generated and the influence of this vascular abnormality to be examined.

### Fluid flow in tumour tissue

#### Effect of vascular distribution and necrosis

**Figure 2:**
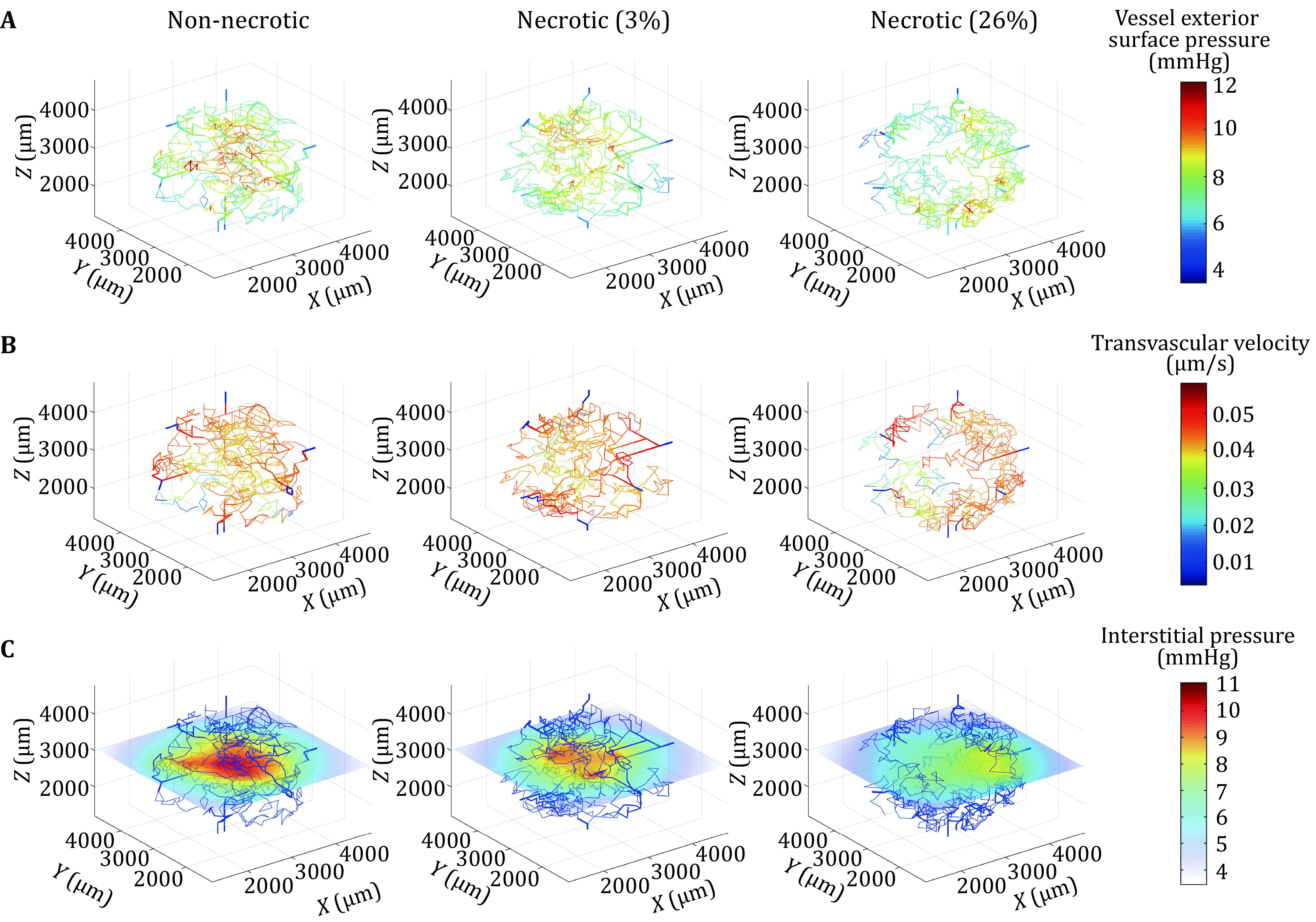
**A** Pressure distribution on exterior wall of vascular network. **B** Transvascular flux distribution. **C** Interstitial pressure distribution in tumour tissue at mid-plane (*z* = 3000 μm)

Intravascular and interstitial pressure distributions across the vascular and interstitial space were calculated as described in the Methods section. Initially, cases of tumours with varying degrees of necrosis as described in Table 1 were evaluated to understand the effect of vascular distribution on fluid flow properties. Figure 2 shows various fluid flow properties calculated for the different tumour models. As shown in Fig. 2A, interstitial pressure at the outer surface of vessels is higher close to the tumour center than in the peripheral region. For the non-necrotic tumour case, the hydrostatic pressure reaches a maximum of approximately 12 mmHg at the surface of some tumour vessels with an average surface pressure of 8.6 ± 1.41 mmHg. Pressures at the surface of vessels in the normal tissue region are much lower (3–4 mmHg), which can be attributed to the low hydraulic conductivities prescribed to these vessels. The predicted interstitial pressures in the normal tissue region are within the range for normal tissues (−8 to 6 mmHg) (Kurbel *et al*. 2001). In the 3% necrotic tumour case, the average interstitial pressure on the surface of the vessels is lower compared to the non-necrotic case, whilst in the tumour with a higher degree of necrosis (26%) the average interstitial pressure on the vessel surface drops even further. The predicted interstitial pressures in the tumour region are within the range of measured values (4–50 mmHg) reported in the literature (Less *et al*. 1992). Figure 2B shows the transvascular flux distribution across the tumour network where a net outward fluid flux is observed in intratumoural vessels, which is significantly higher than for vessels in the normal tissue region where the net fluid flux is close to zero. It also shows that transvascular flux is lower in the central tumour region than in the periphery.

Figure 2C shows the IFP distribution at a mid-height (*z* = 3000 μm) in the tumour tissue. IFP at this location is highest in the central tumour region reaching a peak of 11 mmHg which then drops rapidly moving away from the tumour center where a pressure of around 2.5 mmHg is calculated at the tumour surface. This IFP profile corresponds with experimental and modelling based studies in the literature and further justifies the validity of the model in capturing the flow dynamics in tumour tissue. It is clear that the IFP profile is sensitive to the degree of necrosis, with a pronounced reduction in IFP in the avascular core in the 26% necrotic tumour. This is not so apparent in the tumour with 3% necrosis where high pressures are still seen in the tumour core, but with a slightly lower magnitude compared to the highest pressure found towards the periphery of the tumour. In the 26% necrotic tumour, IFP at the tumour center is significantly lower than the highest pressure distributed at the tumour periphery. The necrotic tumours exhibit pressure profiles that differ from the non-necrotic tumour.

#### Effect of vessel pruning and blind end removal

The vascular networks examined so far feature a number of blind ends that may affect the pressure drop and flow in the vascular network. To quantify this effect, the tumour vascular geometries were modified by removing several blind ends to open up the pathways for flow. To achieve this, the blind ends were identified within the network, which were then either removed or assigned as venous end vessels depending on their order within the network. Likewise, an AV shunt was also placed in a region near the arterial vessels. The macroscopic properties of the tumour microvasculature are similar as seen in Table 2. To quantify the conductivity of the network for blood flow, parameters including mean vascular segment transit times (VSTT) and percentage of hypo-perfused vessels were calculated and compared for the original and pruned networks. As shown in Table 2, removing blind ends leads to a significant reduction in VSTT and the number of hypo-perfused vessels; the latter is reduced by approximately 34%.

**Table 2:** Morphological and hemodynamic parameters for tumour vasculature

Parameter	Unit	Original network	Pruned blind ends network
Morphological parameter			
Vascular density ($ {V}_{{\rm{d}}}) $	%	0.250	0.238
Length density ($ {L}_{\mathrm{D}}) $	mm/mm^3^	10.675	10.246
Surface area to volume ratio (vascular) (*S*/*V*)	mm^2^/mm^3^	216.677	218
Maximum extravascular diffusion distance (*R*)	μm	172.683	176.26
Mean vessel diameter	μm	16.074	16.021
Mean vessel length	mm	0.191	0.192
Hemodynamic parameter			
Mean vascular segment transit time (VSTT)	s	39.591	22.136
Hypo perfused vessels (VSTT > 4 s)	%	70.190	36.182

**Figure 3:**
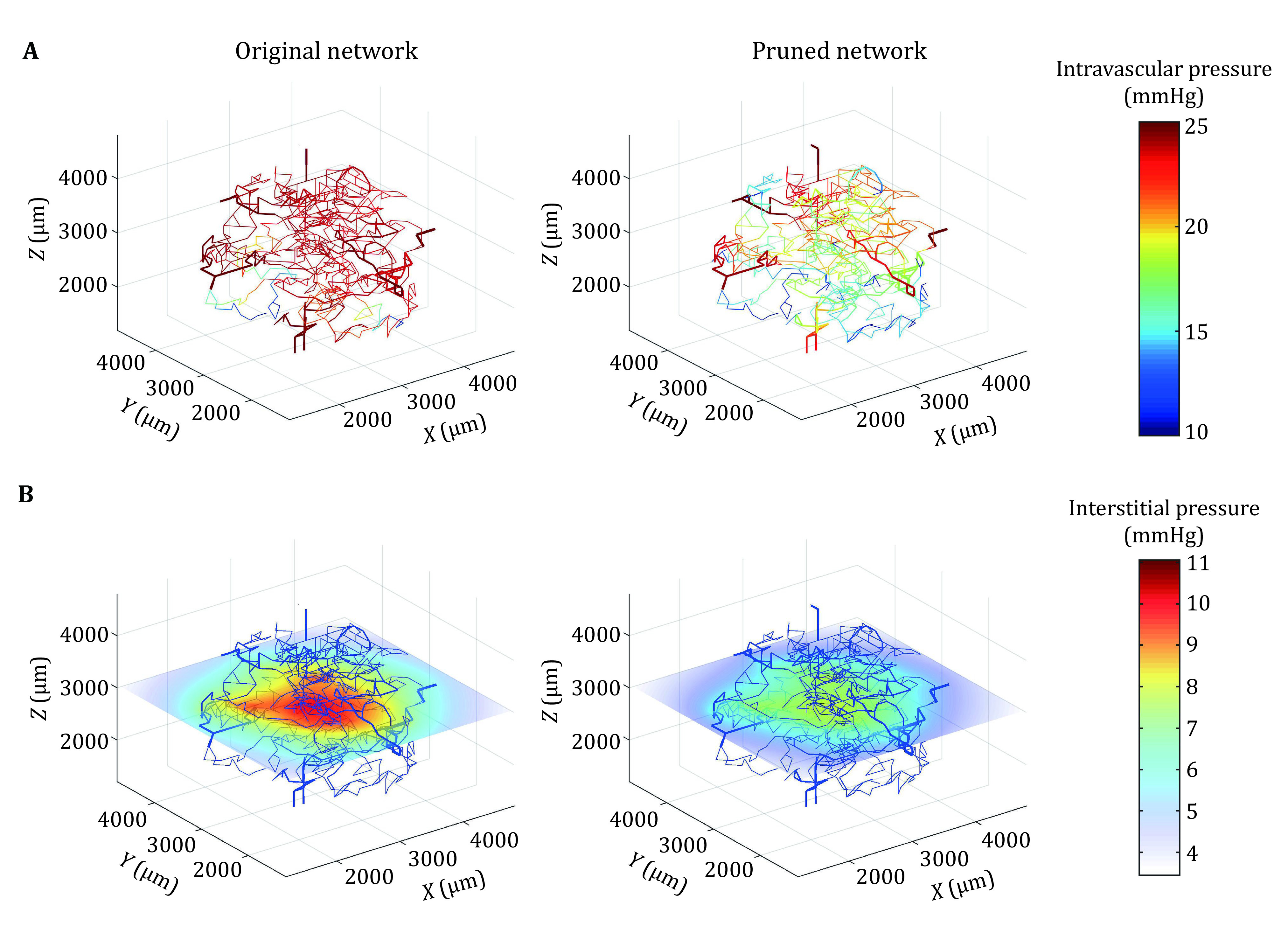
**A** Intravascular pressure in the original and pruned vascular networks. **B** Interstitial pressure in tumour tissue at mid-plane (3000 μm) for the two models

Figure 3A shows the predicted intravascular pressure in the non-necrotic tumour before and after pruning. It is clear that pressure drop along vessels in the pruned vessel network is increased which can promote flow. Figure 3B shows the interstitial pressure distribution at the mid-plane (*z* = 3000 µm), which displays a similar profile but much lower IFP in the pruned network. These findings suggest that although the vascular distribution and macroscopic parameters of the vasculature in the tumour tissue are almost similar between the original and pruned networks, microscopic changes such as the removal of blinds and addition of AV shunts can significantly alter the flow dynamics in the vasculature and consequently in tissue space.

#### Effect of vascular remodelling

**Figure 4:**
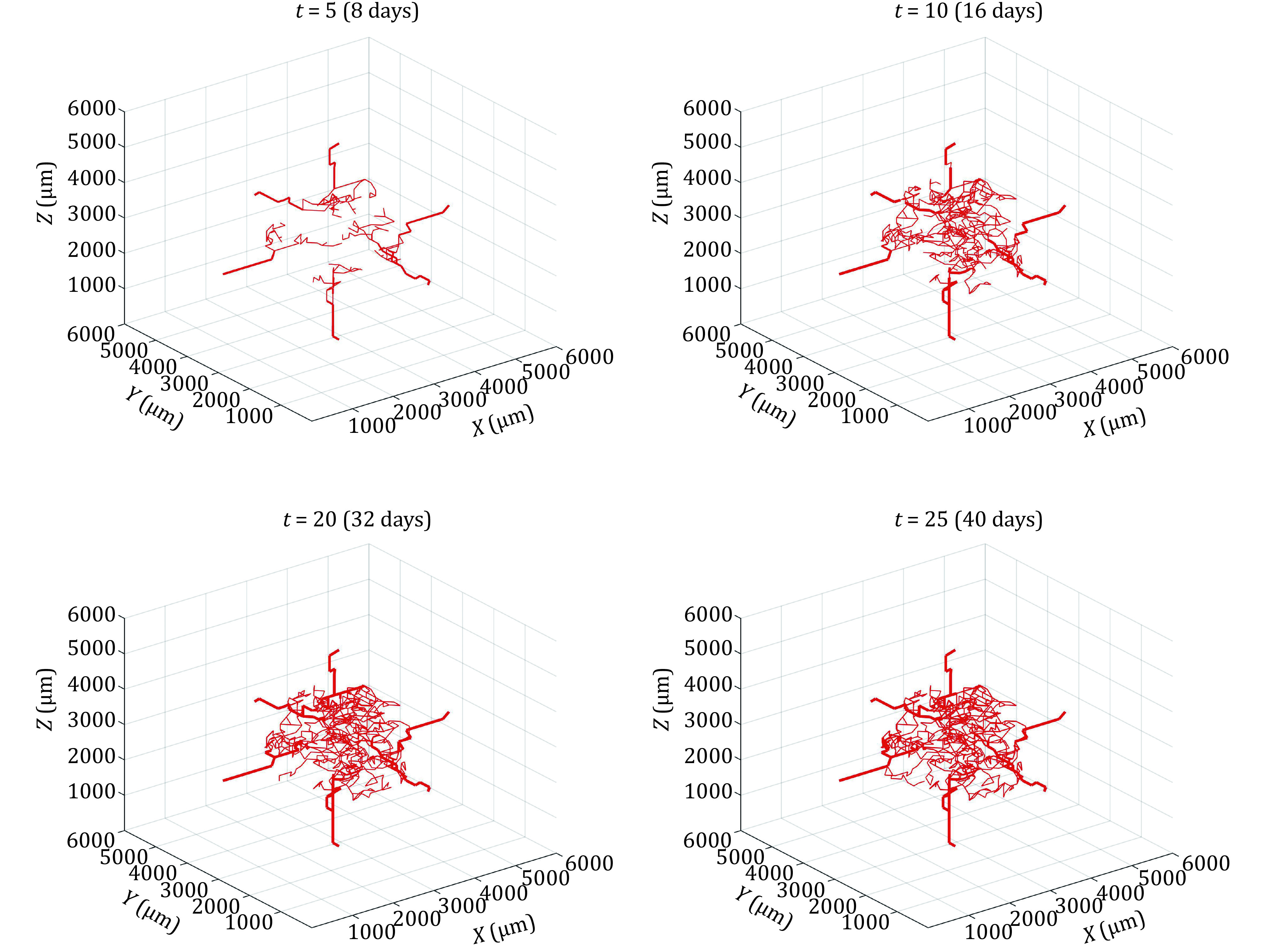
Simulated growth of vascular network with time

To examine the effect of vessel adaption on fluid flow in tumours, the vessel remodelling framework was applied. This was achieved by discretizing the time over which the vascular network grew and calculating the corresponding fluid flow at each time point. Shear stress and time were used as input data to simulate changes in radius, pore size and wall thickness. The evolution of the vascular network with time is shown in Fig. 4.

**Figure 5:**
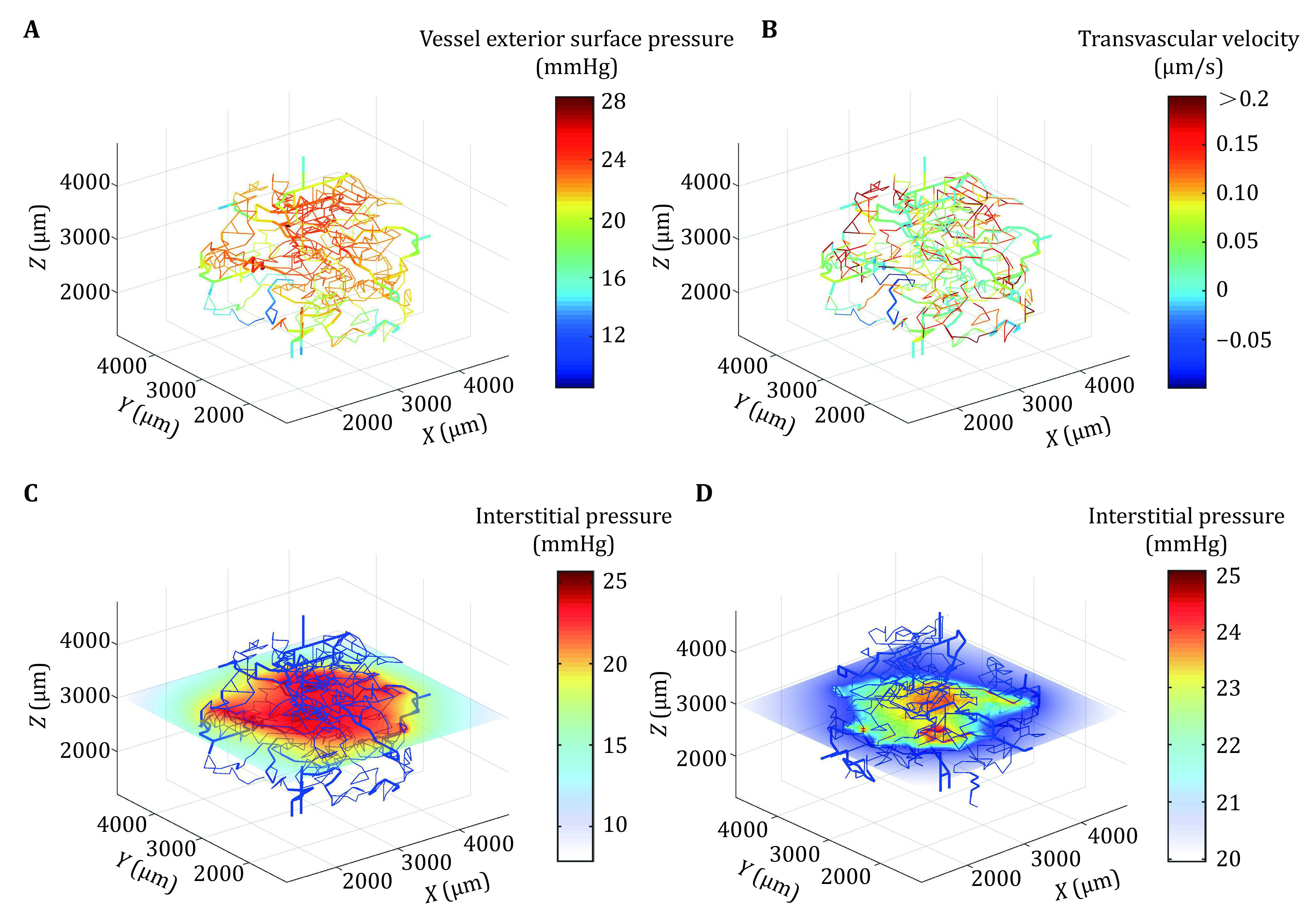
Results for the adaptive tumour network. **A** Vessel surface interstitial pressure. **B** Transvascular flux. **C** Interstitial pressure distribution at mid-plane (*z* = 3000 µm) showing full pressure range. **D** Interstitial pressure in the high range (≥20 mmHg) to show heterogeneity

Figure 5 shows the transvascular flux, vessel surface pressure and interstitial pressure distribution in the adaptive tumour vascular network. The interstitial pressure at the surface of the vessel is seen to be significantly higher compared to the non-adaptive network (Fig. 3), reaching a maximum of around 28 mmHg, which is still within the interstitial pressure values measured in tumours. The transvascular flux is seen to be heterogenous with most vessels exhibiting a flux in the range of 0–0.2 μm/s. Several less mature vessels at tumour center can be seen with an inward flux of <−0.1 μm/s. This is because more recently formed vessels have larger pores and hence the hydraulic conductivity of these vessels is higher. As a result, the IFP is significantly increased in the adaptive network with a maximum pressure of 26 mmHg. In Fig. 5D, IFP in the high range (≥20 mmHg) is displayed to provide a clearer image of the heterogeneity. When a uniform hydraulic conductivity was assumed as shown in Fig. 3, high pressures are mostly concentrated in the central region. The inclusion of adaptive vessel hydraulic conductivity based on vessel maturity and flow dynamics in the tumour leads to a more heterogeneous IFP distribution even in the case of a well vascularized tumour as seen in Fig. 5D.

## DISCUSSION

In this work, a mathematical model was developed where the tumour vascular network is described at a microscopic level on the whole tumour scale and is integrated with Pozrikidis’s fluid flow model to investigate the flow dynamics in tumour tissue. The angiogenesis model was extended to 3D which made it possible to capture the explicit distribution and geometry of the tumour vasculature and its abnormal features. The tumour model was coupled to a fluid flow model which has a distinct advantage of enabling vascular and interstitial flow to be strongly coupled through integration of flow in the vessels and interstitial space over the vessel surface, to provide approximations for fluid flow and transvascular flux that are grounded in physical reality. By applying fluid flow-based boundary conditions on the vessel wall and integrating the pressure over the vessel surface, explicit morphological features of the vasculature including each vessel’s orientation, radius and length can be incorporated. The model was integrated with a vascular remodelling framework that allows for vessel permeability to vary depending on their maturity and local shear stress. The integrated angiogenesis and fluid flow model was evaluated by comparing the obtained morphological and hemodynamic parameters with data in the literature, demonstrating a good agreement.

Using this model, we have been able to demonstrate the strong coupling and interplay between vascular and interstitial flow. Whilst previous studies treating the vasculature as a uniformly distributed source term found IFP to be uniformly elevated across the tumour, in this work we showed that heterogeneous vascular distribution can result in non-uniformly elevated IFP, leading to heterogenous transvascular pressure gradients. Our simulations of fluid flow in tumour vascular geometries with varying degrees of necrosis showed that increasing the necrotic core size resulted in a lower maximum IFP. This finding agrees with a macroscopic study by Soltani and Chen who found that for a small tumour of 1 mm in radius, the presence of a necrotic core as small as 10% of the tumour radius caused a reduction in the maximum IFP (Soltani and Chen 2011). In our case the tumour radius was 1.5 mm. Our results further revealed a non-uniform IFP distribution in necrotic tumours where a dip was seen at the tumour centre which became more apparent with increasing size of the necrotic core. This finding has an important implication for the transport of chemotherapeutics. A dip in pressure at the core would allow for pressure gradients to form from high levels in the tumour periphery to low levels in the necrotic core. For large drugs and liposomes that diffuse slowly, such a pressure gradient would drive convective transport of drugs towards the necrotic region where cells are hypoxic and more resistant or are dead, thus limiting the ability of the drug to target actively proliferating cells. In addition, the large pressure drop at tumour boundary with normal tissue can result in drug clearance from the tumour into the normal tissue, reducing the residence time of the drug in the active tumour regions. This proposition is supported by observation from an experimental study by Vavra *et al*. who investigated the distribution of molecules delivered intravenously in subcutaneous RG-2 tumours (Vavra *et al*. 2004). They found that within 2 h following intravenous administration, the molecules accumulated mainly in the tumour’s boundary and necrotic regions with significantly lower concentrations in the viable tumour region. On the other hand, the transport of intravenously administered drug in non-necrotic tumours might be limited by low extravasation from the blood vessels as evidenced by the lower transvascular flux in the tumour center where IFP is the highest (Fig. 2B). Skliarenko *et al*. demonstrated that higher IFP correlated with enhanced cell survival following treatment with a vascular disrupting agent (Skliarenko *et al*. 2006).

In order to investigate the effect of microscopic features of the vasculature, the flow was analysed in two vascular networks with an almost identical vascular distribution and macroscopic parameters that differed only in the removal of blind ends and addition of AV shunts. These subtle changes in the vascular network opened flow pathways and resulted in a pronounced increase in intravascular pressure drop across the tumour network and a reduction in IFP. This highlights that although vascular networks may appear almost identical from a macroscopic point of view as determined by their vascular density and microvascular distribution, small differences on a microscopic scale can result in dramatically altered flow properties. The ability of normalizing vessel networks has been suggested as a target to enhance treatment (Cantelmo *et al*. 2017; Martin *et al*. 2019). Chauhan *et al*. simulated vessel normalization by reducing the permeability and heterogeneity in vessel permeability, showing that IFP was reduced significantly (Chauhan *et al*. 2012). IFP reduction was also reported by Sweeney *et al*. who investigated vessel normalization by reducing the vessel diameters and regulating vascular hydraulic conductivity values (Sweeney *et al*. 2019). Experimental studies have shown that the treatment of tumours with anti-angiogenic drugs to normalize vasculature resulted in a significant reduction in IFP (Tong *et al*. 2004). Blind ends and self-loops are abnormal features of the tumour vasculature; they cause flow stagnation and reduce perfusion to nearby regions of the tissue. In our work, vascular normalization was simulated by reducing the abnormalities in the architecture of the tumour vasculature by removing blind ends and self-loops. The resultant increase in intravascular pressure drop can promote perfusion and allow intravenously injected drugs to distribute more effectively within the vascular network. In addition, the reduced IFP would allow the drug to permeate through the vessel wall and limit efflux of the drug to the surrounding normal tissue thereby increasing drug residence time.

Finally, the influence of vascular remodelling was examined, which was incorporated by allowing the radius, porosity and wall thickness of each vessel to vary depending on the age and flow properties. Whilst most of the previous studies investigated fluid flow in tumours using a uniform vessel hydraulic conductivity, a vascular remodelling framework was adopted in this study that explicitly defines hydraulic conductivity values for each vessel based on its maturity and perfusion. Our results showed that accounting for vascular remodelling caused a dramatic increase in IFP. The non-uniform vessel permeability led to heterogenous transvascular flux across most of the tumour network. Chauhan *et al*. incorporated a heterogeneous pore size distribution in their model (Chauhan *et al*. 2012), they assigned each vessel a pore size assuming a unimodal distribution throughout the vasculature and hence did not explicitly incorporate the effect of fluid flow or vessel maturity on the permeability of the vessel. Their work showed similar findings where IFP was heterogenous, but it could not be determined from their presented data whether this was due to the heterogeneity of the vessel pore size or the vascular distribution. In the model developed by Vavourakis *et al*., IFP was calculated incorporating heterogenous vessel permeability, however, their model applied the homogenization technique whereby vascular properties were averaged within each element and IFP was evaluated as a function of averaged vascular property parameters within the element (Vavourakis *et al*. 2017). Hence, the morphological effect of the vascular network was not explicitly incorporated when determining IFP. In their work, the tumour IFP values were found to be elevated, however the radial pressure profile was relatively uniform across the tumour before dropping rapidly at the boundary. In our study, by applying a fluid flow model that takes into account the explicit morphology of the vasculature, we found IFP to be more heterogeneously distributed with steeper pressure gradients. The predicted IFP profile in the tissue could also pose an obstacle to the transport of therapeutics with steeper gradients at the tumour periphery and increased interstitial fluid velocity potentially promoting efflux of therapeutics from the tumour tissue. Non-uniform gradients were found across the tumour tissue with IFP peaks being distributed heterogeneously within the tumour. As described previously, high IFP can influence the proliferation of cancer cells and their chemosensitivity, therefore, heterogenous IFP values with steeper gradients could lead to a non-uniform response to chemotherapy in the interstitial space. The non-uniform transvascular flux can result in intravenously administered drugs to extravasate from the vessels at greater concentrations in specific tissue regions and allow other regions to escape treatment.

The angiogenesis model used to describe the tumour vasculature was able to capture the heterogeneous properties of the vasculature and incorporate various aspects of the angiogenesis process. In the model, the tumour was assumed to be of a fixed size and the effect of tumour growth during angiogenesis was neglected. This can be justified as the time scale on which tumour growth occurs is much larger than that of vascular growth. However, changes in oxygen concentration caused by vascular remodelling were not incorporated which can influence the distribution of chemical species secreted which would in turn influence the angiogenic process and vascular network generated. Mechanical forces such as solid stress induced by the growing tumour can affect the movement of endothelial cells which has not been incorporated in the angiogenesis model (Welter and Rieger 2010). The stress caused by proliferating cancer cells can compress and shut down the function of some vessels. These factors could influence the spatial arrangement and orientation of the vascular networks produced (Jain *et al*. 2014). These limitations were not addressed in the current study so as to keep the model computationally tractable. In the fluid flow model, blood was modelled as a continuum Newtonian fluid with a constant viscosity. Blood is in fact a non-Newtonian fluid composed of multiple cellular components, which can have an important influence in small capillaries. Spatial and time variations in these components may result in variable viscosity within the vessel network. Additionally, the response of vessel diameter to changes in transmural pressure and metabolic stimuli has been neglected. Existing models that incorporate these changes were developed using data obtained from vessel networks in normal tissues (Pries *et al*. 2001, 2009). Tumours exhibit a different functional behaviour and would be expected to respond differently. When considering interstitial flow, the tissue was assumed to be a homogenous porous medium. In reality, tumour tissue is heterogeneous with varying porosity and interstitial hydraulic conductivity which consequently affect fluid flow. Further research can be built on this model to incorporate tissue heterogeneity and more complex angiogenesis models.

## CONCLUSIONS

Previous models that assume a uniform vascular distribution or treat the vasculature as a uniform source term do not capture the effect of neighbouring vessels on each other, which could potentially influence flow on the macroscopic scale. By using the boundary integral method to distribute sources and dipoles along the vessel surface, our model allows for the topology and geometry of the vasculature to be taken into account, so that the effect of each vessel segment on IFP at any point in space is considered. The model also accounts for the influence of IFP on intravascular flow, in an attempt to provide a more realistic prediction of fluid flow under tumour physiological conditions. Using this method, the work presented in this paper has demonstrated the importance of considering not only the explicit nature of the tumour vasculature but also the intravascular flow properties when predicting and evaluating fluid flow in tumour tissue. Our results shed further light on the intricate flow dynamics occurring on a microscopic scale in the tumour tissue and their potential consequences on macroscopic flow and drug transport. Our model can be further extended to predict drug distribution in tumour tissue, and this will be reported separately.

## METHODS

### Tumour model

#### Tumour induced angiogenesis

The tumour model is generated to provide an explicit representation of the tumour vasculature at a microscopic scale. The extravascular space is assumed to consist of uniformly distributed cancer cells and extracellular material. To generate the explicit tumour vasculature, Anderson and Chaplains mathematical angiogenesis model is implemented (Anderson and Chaplain 1998). The model describes the movement of endothelial cells through random motility, chemotaxis in response to tumour angiogenic factor gradients (TAF) and haptotaxis in response to fibronectin. The original 2D angiogenesis model has been extended to 3D to describe the movement of endothelial cells along each orthogonal axis in a 3D space. The non-dimensional equations, endothelial cell density (*n*), fibronectin concentration (*f*) and tumour angiogenic factor concentration (*c*), which describing vascular growth are given below:

1 $
\frac{\partial n}{\partial t}=D{\nabla }^{2}n-\chi \left(c\right)n\nabla c-\nabla \left(\rho n\nabla f\right)\;, 
$

2 $
\frac{\partial f}{\partial t}=\beta n-\gamma nf\;,
$

3 $
\frac{\partial c}{\partial t}=-\eta nc\;,
$

where $ D $, $ \chi  $ and $ \rho  $ are coefficients for endothelial cell motion, chemotaxis and haptotaxis respectively. $ \beta  $ and $ \gamma  $ are fibronectin production and consumption rates whilst $ \eta  $ is the TAF production rate. These parameters were obtained from Anderson and Chaplain’s work. Time is normalized using the timescale for TAF to diffuse from the tumour to the parent vessel (Anderson and Chaplain 1998). Assuming the cells remain within the boundary and applying a no-flux boundary condition, the discretized form of equations is given by six-point stencil scheme as follows:

4 $
\left\{ 
\begin{aligned} 
n_{l,m,w}^{q + 1} =\; &n_{l,m,w}^q{P_0} + n_{l + 1,m,w}^q{P_1} + n_{l - 1,m,w}^q{P_2} + n_{l,m + 1,w}^q{P_3} \\&+ 
n_{l,m - 1,w}^q{P_4} + n_{l,m,w + 1}^q{P_5} + n_{l,m,w - 1}^q{P_6}\;,\\
f_{l,m,w}^{q + 1} =\; &f_{l,m,w}^q\left[ {1 - k\gamma n_{l,m,w}^q} \right] + k\beta n_{l,m,w}^q\;,\\
c_{l,m,w}^{q + 1} = \;&c_{l,m,w}^q\left[ {1 - k\eta n_{l,m,w}^q} \right]\;,

\end{aligned} \right.
$

where subscripts $l,\;m \;{\rm{and}}\;w$ denote position on the grid and $ q $ denotes dimensionless timestep.

Initial conditions are defined for a spherical tumour centred in the domain with a non-dimensionless radius of 0.25 surrounded by normal tissue. Hence, TAF concentration is expected to be highest in the tumour region as it is secreted by tumour cells with concentration decreasing as distance from the tumour increases. Initial TAF concentration $ c $ is defined as follows:

5 $
\left\{ \begin{array}{l}
c\left({x,y,z,0} \right) = \left\{ {\begin{array}{*{20}{c}}
{1,\;\;\;\;\;\;\;\;\;\;r < 0.25}\\
{\dfrac{{{{(v - r)}^2}}}{{v - 0.25}},\;r \ge 0.25}
\end{array}} \right.,\\
r = \sqrt {{{(x - 0.5)}^2} + {{\left({y - 0.5} \right)}^2} + {{(z - 0.5)}^2}}\;\;,
\end{array} \right.
$

where $ v $ is a positive constant and $ r $ is the normalized distance from the tumour center assuming the tumour is centred at (0.5, 0.5, 0.5) within the domain. A parent vessel is assumed to exist at the center of each face of the domain. The initial distribution of endothelial cells *n* describes the location of the parent vessels within the domain. The cells are assumed to form clusters at each face of the domain where the parent vessels are located.

Fibronectin is assumed to be highest in regions in and around parent vessels. Anderson and Chaplain justified this by referring to experimental observations on increased extracellular molecules around parent vessels (Anderson and Chaplain 1998). This relationship is adapted in our model and is described by the following:

6 \begin{equation*}\begin{split} 
f\left({x,y,z,0} \right) =\;& 0.35\left(k{e^{ - \frac{{ - {x^2}}}{{{\epsilon_2}}}}} +\; k{e^{ - \frac{{ - {{\left({x - 1} \right)}^2}}}{{{\epsilon_2}}}}} \right.\\&\left.+\; k{e^{ - \frac{{ - {y^2}}}{{{\epsilon_2}}}}} +\; k{e^{ - \frac{{ - {{\left({y - 1} \right)}^2}}}{{{\epsilon_2}}}}} +\; k{e^{ - \frac{{ - {z^2}}}{{{\epsilon_2}}}}} +\; k{e^{ - \frac{{ - {{\left({z - 1} \right)}^2}}}{{{\epsilon_2}}}}} \right).
\end{split}\end{equation*}

A tumour with a necrotic core of radius 0.08 can be generated by simulating a negative TAF gradient at the center with a defined size as follows:

7 $
c\left({x,y,z,0} \right) = \left\{ {\begin{array}{*{20}{l}}
{0.9,}&{r \le 0.04,}\\
{0.8 + 2.5r,}&{0.04 \le r \le 0.08,}\\
{1,}&{0.08 \le r \le 0.25,}\\
{\dfrac{{{{\left({v - r} \right)}^2}}}{{v - 0.25}},}&{r \ge 0.25.}
\end{array}} \right.
$

**Figure 6:**
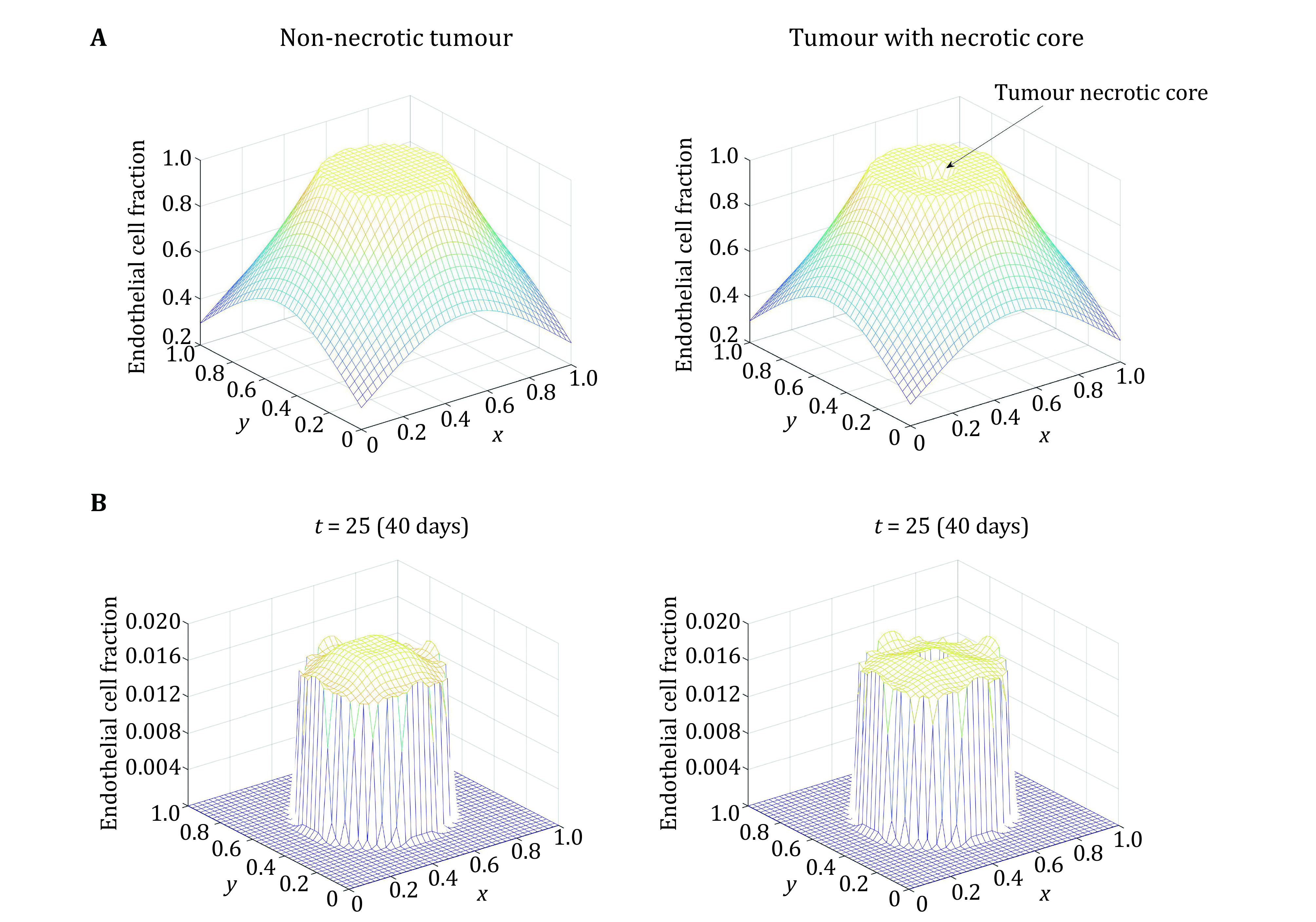
**A** Initial endothelial cell distribution. **B** Endothelial cell distribution after 40 days

The endothelial cells are unlikely to move down the concentration gradient of TAF. Eq. 5 and 6 are used to initialise the model and a spatiotemporal evolution of the endothelial cell distribution is obtained using Eq. 4. Figure 6A shows the initial TAF distribution for the non-necrotic and necrotic core tumours. Figure 6B shows the spatio-temporal distribution of endothelial cells where the concentration is zero in the necrotic region defined.

**Figure 7:**
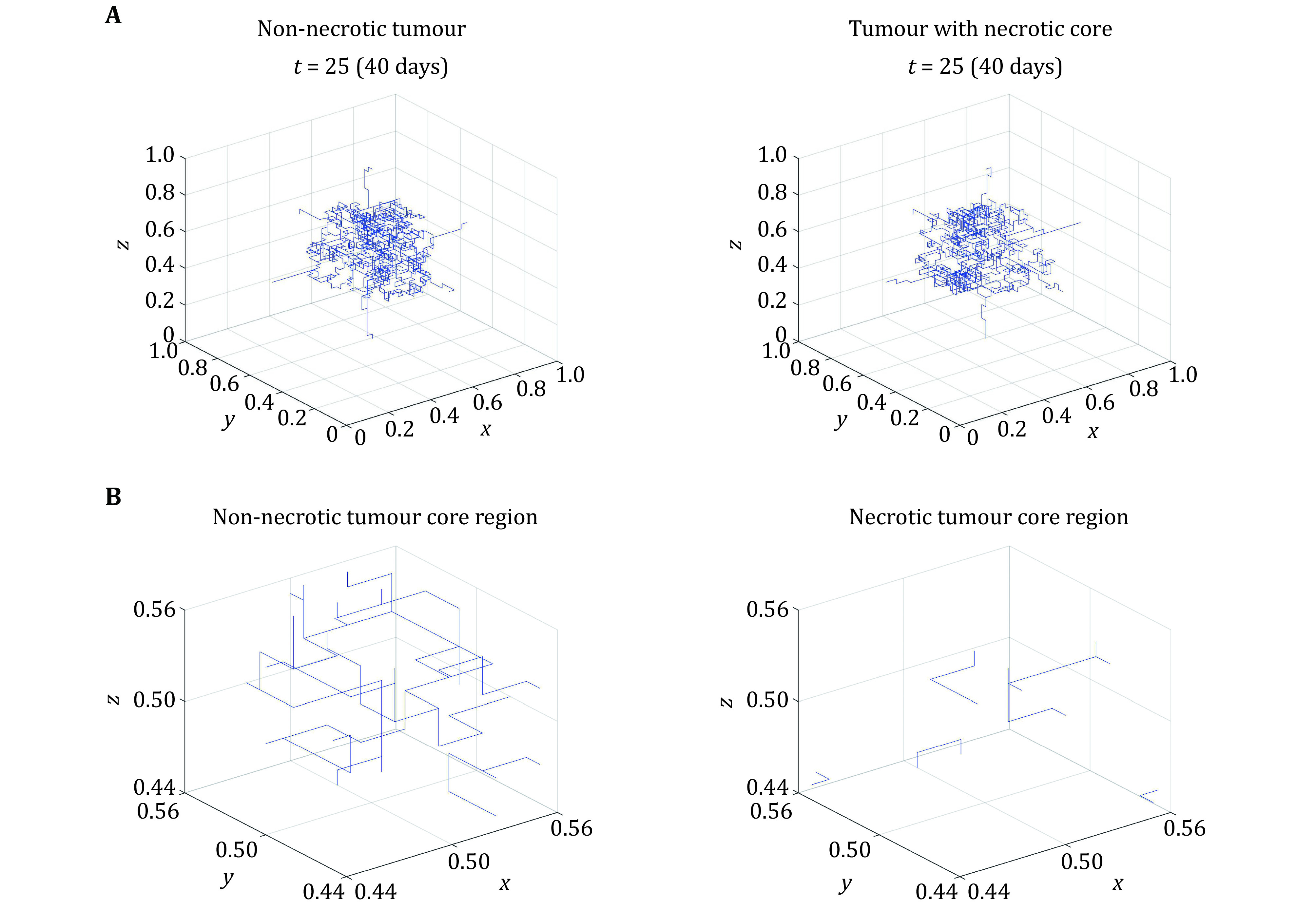
**A** Capillary network formed after 40 days for a non-necrotic and necrotic tumour. **B** Core region of non-necrotic and necrotic tumour

The endothelial cell distribution field is combined with a discrete model to track the movement of tip endothelial cells and resulting vascular network formed. The migration of the tip endothelial cells is determined by seven probability coefficients P_0_–P_6_ which correspond to the probability of no movement (P_0_), movement along the *x*-axis (P_1_,P_2_), movement along the *y*-axis (P_3_,P_4_), and along the *z*-axis (P_5_,P_6_). The magnitude of the probability coefficients depends on local TAF and fibronectin concentrations. At each time step the seven probability coefficients are calculated to produce seven ranges between each coefficient. A random number is generated between 0 and 1 and the movement of the endothelial cell depends on the range where the random number falls. Hence, the movement of the tip endothelial cell is based on three components: random, chemotactic and haptotactic movement. Branching and anastomosis are common features in tumour vasculature, and these are represented following the approach of Anderson and Chaplain (Anderson and Chaplain 1998) which is summarized here. Branching from a sprout is dependent on three factors: (1) branching age — a sprout must reach a mature state, measured by a threshold branching age, for it to branch; (2) space — there must be space for the sprout to branch into, checked by ensuring there are no other sprouts around the tip; (3) the number of endothelial cells at the tip — an endothelial cell density threshold must be satisfied for the sprout to branch. If the above three conditions are met, the branching of a sprout is given as a probability that is dependent on the local TAF concentration. The higher the TAF concentration, the greater the chances are for a sprout to branch. Overall, this model describes the movement of a sprout tip from the parent vessels where there is little branching due to the age and as the sprouts reach the tumour, the increased age combined with the high TAF concentration increases the chance of vessel sprouting. Anastomosis or merging of vessels is described simply when a sprout moves into a space occupied by another sprout. The sprout that continues to exist is chosen at random. Combining the discrete method with the fields generated for endothelial cell density, TAF and fibronectin, vascular network geometries can be generated as seen in Fig. 7. Figure 7B highlights the avascular core region in the necrotic tumour that results from the presence of a negative TAF gradient at the tumour core.

#### Vascular network regulation

**Figure 8:**
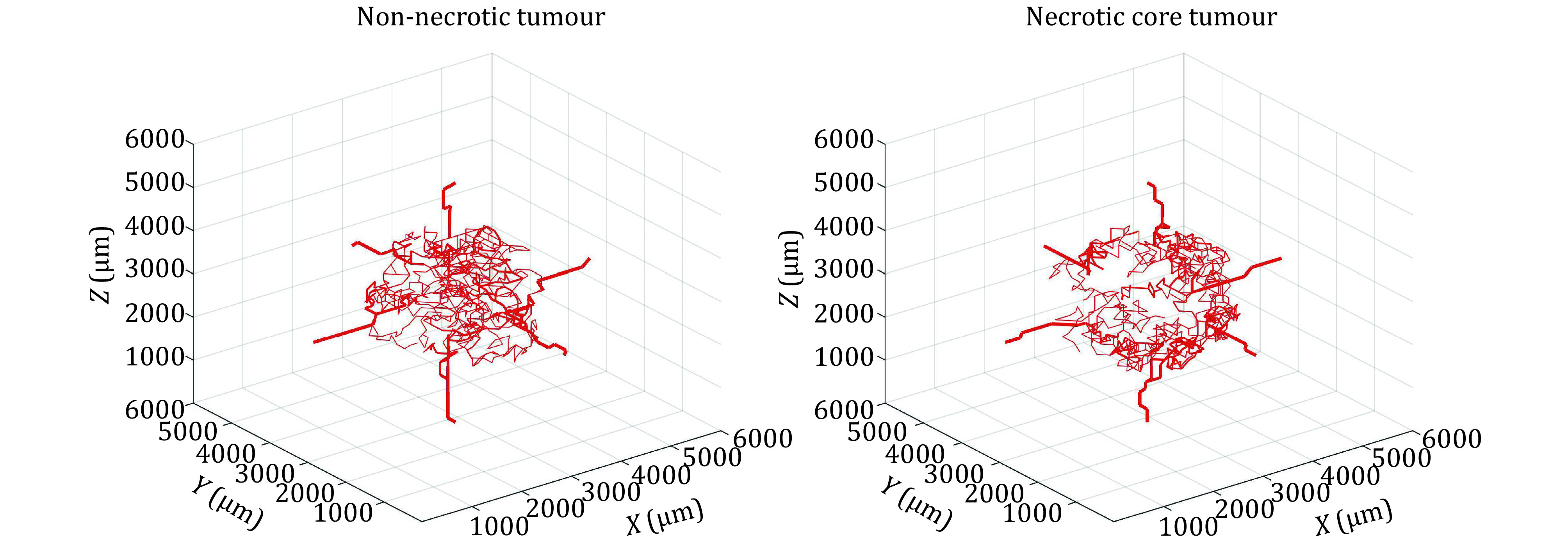
Generated capillary network for non-necrotic and necrotic tumour

In order to apply the fluid flow model to the vascular network generated from the angiogenesis model, the geometry of the network must be regulated and further defined. First, nodes are assigned to coordinates where tip merging or branching occurs and where the tips initially sprout and end. Then further nodes are created along the path that the tip moves. A connection matrix logging the connectivity between the nodes is formed based on the data from the raw vascular network generated. A depth-first search algorithm is then used to move down from the initial node, creating segments based on the connection matrix. To define the diameter of the vessels, a maximum diameter is set at the initial parent vessels which are assumed to be maintained for subsequent vessels down the network, except in the case where branching or merging occurs. When this occurs the diameter of the daughter vessel is assumed to decrease in size. The change in radius from a vessel of the *n*^th^ generation to the (*n*+1)^th^ generation can be modelled as monotonously decreasing as follows:

8 $
{D}_{n+1}={{\left(\frac{{D}_{min}}{{D}_{max}}\right)}^{m}D_{n}} 
$

where $ {D}_{min} $ and $ {D}_{max} $ are defined minimum and maximum diameters, and *m* is a coefficient less than 1. Applying the vascular regulation algorithm to the networks generated from the angiogenesis model yields the vascular geometries shown in Fig. 8.

#### Vascular remodelling framework

Blood vessels in tumour tissues are characterized by their heterogeneous nature where they can exhibit varying diameters and permeabilities. Blood vessels naturally adapt in response to mechanical and metabolic stimuli in order to ensure the tissue is well perfused and supplied with nutrients and oxygen. The mechanisms of vessel adaption have been described by Pries *et al*., which provides a framework to model changes in vascular structure in response to stimuli (Pries *et al*. 2001). The parameters in their model were fitted specifically to the normal tissue which was studied in their work. Cellular abnormalities in the tumour vessels such as large inter-endothelial gaps limit signal conduction, whilst the lack of pericyte coverage reduces the ability to undergo diameter changes. The impaired structural adaption in addition to the lack of experimental data on tumour vessels makes the Pries model less applicable in this case. Hence, a simple framework is employed in this study, which was developed by Vavourakis *et al*. to model changes in vessel radius, wall thickness and pore radius (Vavourakis *et al*. 2017). In their work, vessel adaptivity is described by a single variable, the remodelling time $ {t}_{m} $ which is governed by the shear stress on the vessel and is given by Eq. 9:

9 $
 
{t_m}(\tau  ) =
\left\{ {\begin{array}{*{20}{l}}
{{t_{m - T}},}\;\;\;\qquad\qquad\qquad\qquad{\tau  \ge {\tau _{ref,}}}\\
{t_{m - T}} + ({{t_{m - 0}} - {t_{m - T}}} )\cdot\\\qquad{\rm{exp}}[ {1 - {{({1 - {\tau ^2}/\tau _{ref}^2} )}^{ - 1}}} ],\;\;\;{\tau  < {\tau _{ref,}}}
\end{array}} \right.

$

where $ {t}_{m-T} $ is the time value for a vessel to reach the upper limit of remodelling if $  \tau \ge {\tau }_{ref} $, and $ {t}_{m-0} $ is the time required for vessel remodelling if shear stress is zero. Here, we set $ {t}_{m-0} $ to 100 d and $ {t}_{m-T} $ to 10 d based on the empirical value adopted by Vavourakis *et al*. (Vavourakis *et al*. 2017). The model assumes that a vessel becomes fully remodelled as it moves from a poorly perfused state to a well perfused state which is defined by the reference shear stress, $ {\tau }_{ref} $. Vascular segment transit time (VSTT) is used as an indicator for hypo perfusion as it takes into account blood flow and vessel length in each segment. Kamoun *et al*. defined hypo-perfused vessels as those where velocities are below 0.05 mm/s, hence for an average vessel of 0.2 mm in length, the VSTT is approximately 4 s (Kamoun *et al*. 2010). The reference shear stress can be evaluated in terms of VSTT as follows:

10 $
{\tau }_{ref}=\frac{4\mu L}{\mathrm{V}\mathrm{S}\mathrm{T}\mathrm{T}\mathrm{ }\cdot R}\;\;.
$

In vessel adaption model developed by Vavourakis *et al.*, the change in capillary radius is modelled as a function of normalised time, $\tilde t =\dfrac{t-{t}_{i}}{{t}_{m}}$, where $ {t}_{i} $ is the time point at which the vessel is generated and $ {t}_{m} $ is the remodelling time which is a function of shear stress as described in Eq. 9. Initially when a node is created through the movement of the vessel tip, the vessel is assigned a minimum radius $ {R}_{min } $ which then expands with time and as the shear stress increases. The change in radius is defined using the following expression:

11 $

R\left({\tilde t} \right) =
\left\{ 
\begin{aligned} &
{{R_{min}},}\;\;\;\;\qquad\qquad\quad\qquad\qquad{{\rm{at}}\;{\rm{tip}}\;{\rm{node}},}\\&
{R_{min}} + ({R_{max}} - {R_{min}})\cdot\\&\qquad{\rm{exp}}\left[ { - 11{\rm{exp}}\left({ - 4.4\tilde t} \right)} \right],\;\;\;\;{{\rm{elsewhere}},}

\end{aligned}\right.

$

where $ {R}_{max} $ is the maximum radius. Wall thickness, $ w $, can be assumed to increase linearly as a function of time and is given as

12 $
w\left({\tilde t} \right) = {w_{max}} + \left({1 - \tilde t} \right){w_{min}}
$

where $ {w}_{max} $ and $  {w}_{min} $ are the maximum and minimum wall thickness. The vessel pore size is also modelled as a function of time and shear stress and is expressed as

13 $

{r_p}\left({\tilde t} \right) =
\left\{     {\begin{array}{*{20}{l}}
{{r_{pmin}},}\;\;\;\;\;\;\qquad\qquad\qquad\qquad\qquad{\tilde t \ge 1,}\\
2\left({{r_{pmax}} - {r_{pmin}}} \right){{\tilde t}^3} \\ \quad+ 3\left({{r_{pmin}} - {r_{pmax}}} \right){{\tilde t}^2} + {r_{pmax}},\;\;{{\rm{elsewhere}},}
\end{array}} \right.

$

where $ {r}_{pmin} $ and $ {r}_{pmax} $ are the minimum and maximum pore size respectively. Initially when a vessel is generated, it is assigned a radius $ {R}_{min} $, pore size $ {r}_{pmax} $ and wall thickness $ {w}_{min} $. This follows physiological behaviour as when a vessel initially sprouts and is in an immature state, it is highly porous with thin walls. The hydraulic conductivity of each vessel is determined as a function of wall thickness $  w $, pore radius $ {r}_{p} $ and the fraction of vessel wall occupied by pores $  {\gamma }_{p}: $

14 $
{L_p} = \frac{{{\gamma _{p}}r_p^2\left({\tilde t} \right)}}{{8\mu w\left({\tilde t} \right)}}.
$

### Fluid flow model

Fluid flow in the tumour model can be described in three parts, vascular flow, transvascular flux and interstitial flow. Blood flow is modelled using Poiseuille’s law which describes flow rate, $  Q $, through a straight cylindrical segment as a function of radius $ R $ and pressure drop. Transvascular flux, $ {q}_{e} $, is described by starling’s filtration law whilst interstitial flow is described by Darcy’s law through porous medium.The governing equations used to model these flows are summarised as follows:

blood flow:

15 $
Q\left(l\right)=-\frac{\pi {R}^{4}\left(l\right)}{8\mu }\frac{d{p}_{v}}{dl},
$

transvascular flux:

16 $
{q}_{e}\left(l\right)={L}_{p}\left(l\right)\left[{p}_{v}\left(l\right)-{p}_{i}\left(l\right)\right],
$

interstitial flow:

17 $
{u}_{i}=-\kappa \nabla {p}_{i}\;,
$

where $ {p}_{v} $ and $ {p}_{i} $ are vascular and interstitial pressures respectively, $ \mu  $ is the blood viscosity, $ l $ is distance along vessel segment, $ {u}_{i} $ is the interstitial fluid velocity and $ {L}_{p} $ and $ \kappa  $ are vascular and interstitial hydraulic conductivities respectively. Applying mass conservation in the interstitial space using Eq. 17 and assuming that $ \kappa  $ is constant, the interstitial pressure satisfies Laplace’s equation,

18 $
\left\{ 
 \begin{aligned} &
{\nabla \cdot u}_{i}=0, \\&
\nabla  \cdot \left({ - \kappa \nabla {p_i}} \right) = 0,\\&
{\nabla ^2}{p_i} = 0.

\end{aligned} \right.
$

Applying Pozrikidis’s method (Pozrikidis and Farrow 2003), flow is assumed to be conserved along the vessel length to couple vascular and interstitial flow,

19 $
\frac{dQ}{dl}+2\pi R\left(l\right){q}_{e}\left(l\right)=0. 
$

Laplace's Eq. 18 for interstitial pressure is reformulated using the boundary integral formulation as in Pozrikidis’s work to give the interstitial pressure at field point $ {\boldsymbol{x}}_{0} $ in terms of a combination of a single- and a double-layer potential defined over the surface of the vasculature. The point $ {\boldsymbol{x}}_{0} $ is set at the surface of vasculature and the interstitial pressure is assumed to be independent of angular pressure giving,

20 \begin{equation*}\begin{split} 

\frac{1}{2}[{p}_{i}({\boldsymbol{x}}_{0})-{p}_{0}]=&\int {\int }_{SV}^{PV}[{p}_{i}(l)-{p}_{0}][\mathrm{n}(\boldsymbol{x})\nabla G(\boldsymbol{x},{\boldsymbol{x}}_{0})]dS(\boldsymbol{x})\\&+\int {\int }_{SV}\frac{{L}_{p}(l)}{\kappa }[{p}_{v}(l)-{p}_{i}(l)]G(\boldsymbol{x},{\boldsymbol{x}}_{0})dS(\boldsymbol{x}),

\end{split}\end{equation*}

where $ {p}_{0} $ is the pressure at the tumour surface, $  G\left(\boldsymbol{x},{\boldsymbol{x}}_{0}\right) $ is the Green’s function solution to Laplace’s equation that is dependent on the tumour geometry and $  S\left(\boldsymbol{x}\right) $ denotes the tumour surface boundary.

Substituting Eq. 15 and 16 into Eq. 19 and assuming a constant radius for each vessel segment gives

21 $
\frac{{d}^{2}{p}_{v}}{d{l}^{2}}=-\frac{16\mu }{{R}^{3}\left(l\right)}{L}_{p}\left(l\right)\left[{p}_{v}\left(l\right)-{p}_{i}\left(l\right)\right].
$

### Numerical methods

The vasculature is discretized into $ {N}_{v} $ cylindrical segments where *j* = 1, 2, 3, ···, $ {N}_{v} $ and each vessel assigned a radius $ R $, length $ L $ and vascular hydraulic conductivity $  {L}_{p}^{j}. $

For a single vessel with uniform radius, Eq. 21 is discretized using the second order finite difference method as in Pozrikidis’s work to obtain the midpoint vascular pressure in terms of the interstitial pressure and end-point vascular pressures,

22 $
\left\{ 
 \begin{aligned} 
&
p_v^{(j + 1)/2} = \frac{{p_v^j + p_v^{j + 1} + \beta p_i^{j + \frac{1}{2}}}}{{2 + \beta }},\\&
\beta  = 4\frac{{\mu {L_p}{L^2}}}{{{R^3}}}.

\end{aligned}\right.
$

Applying the second order finite difference methods for the first derivative in Eq. 21 gives the pressure gradient at the end nodes of vessel segment *j*:

23 $
\left\{ 
\begin{aligned} 
 &
\left({\frac{{d{p_v}}}{{dx}}} \right)_{{E_j}}^j \cong \frac{{ - \left({3\beta  + 2} \right)p_v^j + 4\beta p_i^{j + \frac{1}{2}} + \left({2 - \beta } \right)p_v^{j + 1}}}{{\left({2 + \beta } \right)L}},\\&
\left({\frac{{d{p_v}}}{{dx}}} \right)_{{E_j}}^{j + 1} \cong \frac{{ - \left({2 - \beta } \right)p_v^j - 4\beta p_i^{j + \frac{1}{2}} + (3\beta  + 2)p_v^{j + 1}}}{{\left({2 + \beta } \right)L}}.

\end{aligned} \right.
$

Combining Eq. 23 with Poiseuille’s law, Eq. 15, the blood flowrate at each segment end can be evaluated as follows:

24 $
\left\{ 
\begin{aligned} &
Q_{{E_j}}^j  =  \left({ - \frac{{d{p_v}}}{{dx}}} \right)_{{E_j}}^j\frac{{\pi {R^4}}}{{8\mu }}  =  \left({{c_A}p_v^j  -  {c_C}p_i^{j + \frac{1}{2}} - {c_B}p_v^{j + 1}} \right)\frac{{\pi {R^4}}}{{8\mu L}},\\&
Q_{{E_j}}^{j + 1}  =  \left({ - \frac{{d{p_v}}}{{dx}}} \right)_{{E_j}}^{j + 1}\frac{{\pi {R^4}}}{{8\mu }}  =  \left({{c_B}p_v^j  +  {c_C}p_i^{j + \frac{1}{2}}  -  {c_A}p_v^{j + 1}} \right)\frac{{\pi {R^4}}}{{8\mu L}},

\end{aligned}\right.
$

here

$
{c}_{A}=\frac{\left(2+3\beta \right)}{2+\beta }, \;\; {c}_{B}=\frac{\left(2-\beta \right)}{2+\beta } \; \; {\rm{and}}\;\;  {c}_{C}=\frac{4\beta }{2+\beta }\;.
$

Mass conservation is assumed in nodes shared between two segments:

25 $
{Q}_{{E}_{j}}^{j+1}={Q}_{{E}_{j+1}}^{j+1}.
$

Combining Eq. 24 with Eq. 25 and rearranging provides a tridiagonal system of equations for the capillary pressure:

26 $
{p}_{v}^{j+1}\left({c}_{A}^{\left(1\right)}+{c}_{A}^{\left(2\right)}\right)-{c}_{B}^{\left(1\right)}{p}_{v}^{j}-{c}_{B}^{\left(2\right)}{p}_{v}^{j+2}{-c}_{C}^{\left(1\right)}{p}_{i}^{j+\frac{1}{2}}{-c}_{C}^{\left(2\right)}{p}_{i}^{j+\frac{3}{2}}=0,
$

for *j* = 2, 3, 4, ···, $  {(N}_{v}-1) $ where $  r = {L_j}/{L_{j + 1}} $. The right hand side is the interstitial pressure on the surface of the vessel; for *j* = 1 and *j* = $ {N}_{v} $, boundary conditions are applied where ${p}_{v}^{1}={p}_{{\rm{arterial}}}$ and ${p}_{v}^{{N}_{v}}={p}_{{\rm{venous}}}$.

In our model with complex vasculature that includes branching and merging, mass conservation at the bifurcating node is applied as follows:

27 \begin{equation*}\begin{split} &
p_v^{j + 1}\left({c_A^{\left(1 \right)} + c_A^{\left(2 \right)} + c_A^{\left(3 \right)}} \right) - c_B^{\left(1 \right)}p_v^j - c_B^{\left(2 \right)}p_v^{j + 2}\\&\qquad
 -  c_B^{\left(3 \right)}p_v^{j + 3} - c_C^{\left(1 \right)}p_i^{j + \frac{1}{2}} - c_C^{\left(2 \right)}p_i^{j + \frac{3}{2}} - c_C^{\left(3 \right)}p_i^{j + \frac{5}{2}} = 0.

\end{split}\end{equation*}

Eq. 20 is discretized where the evaluation point is set to the midpoint of vessel segment and reformulated to give the following:

28 \begin{equation*}\begin{split} &
-\frac{{L}_{p}{A}^{j}}{k\left(2+\beta \right)}{p}_{v}^{j+1}-\frac{{L}_{p}{A}^{j}}{k\left(2+\beta \right)}{p}_{v}^{j}\\&\qquad
+ \left[-{B}^{j}+2\frac{{L}_{p}{A}^{j}}{k\left(2+\beta \right)}\right]{p}_{i}^{j+\frac{1}{2}}=\frac{1}{2}{p}_{0}-{A}^{j}{p}_{0},
\end{split}\end{equation*}

where $ {A}^{j} $ and $ {B}^{j} $ are single and double layer influence coefficients.

Eq. 26, 27 and 28 provide a linear system of equations which is solved subject to arterial and venous boundary pressures and interstitial tumour surface pressure. Initially, inlet and outlet capillary nodes are assigned arterial and venous pressure *p*_arterial_ and *p*_venous_ respectively, while interstitial pressures at the surface of the capillary midpoints are set to $ {p}_{0} $. Eq. 26, 27 and 28 are used to generate a tridiagonal system of equations which is solved in MATLAB to calculate nodal capillary pressures and interstitial pressure at the surface of the vessel segment.

Once the nodal capillary pressures and the interstitial pressure at the surface of the segments are determined, the interstitial pressure in the tissue space is solved using the discretized boundary integral formulation:

29 \begin{equation*}\begin{split} 
\frac{1}{2}\left[{p}_{i}\left({l}_{0}\right)-{p}_{0}\right]=&
{\sum }_{j=1}^{{N}_{v}}\frac{{L}_{p}^{j}}{\kappa }({p}_{v}^{j}-{p}_{i}^{j}){A}^{j}\left({l}_{n}^{m}\right)\\&+{\sum }_{j=1}^{{N}_{v}}({p}_{i}^{j}-{p}_{0}){B}^{j}\left({l}_{n}^{m}\right).
\end{split}\end{equation*}

The interstitial space is discretized and the single- and double-layer potentials $ {A}^{j} $ and $ {B}^{j} $ are used to evaluate the influence of vessel segments on each discretized tissue point. Using the influence coefficients, vessel topology, nodal capillary pressure and interstitial pressure at the vessel surface, the contribution of the vessel towards the pressure in the interstitial space at point $ \boldsymbol{x} $ is calculated, where $ \boldsymbol{x} $ = (*x*, *y*, *z*). The interstitial pressure at point $ \boldsymbol{x} $ can then be calculated as a sum of these contributions and the influence of the tumour surface pressure. Rearranging Eq. 22 gives a formulation for the transmural pressure term $  ({p}_{v}^{j}-{p}_{i}^{j}) $ and is substituted into Eq. 29 to give the interstitial pressure at point $ \boldsymbol{x} $ as follows:

30 \begin{equation*}\begin{split} 
\frac{1}{2}{p}_{i}\left(\boldsymbol{x}\right)=&\sum \limits_{\boldsymbol{j}=1}^{{\boldsymbol{N}}_{\boldsymbol{v}}}{B}^{j}\left({l}_{n}^{m}\right)\left({p}_{i}^{j+\frac{1}{2}}-{p}_{0}\right)\\&+
{A}^{j}\left({l}_{n}^{m}\right)\frac{{L}_{p}}{\kappa }\frac{{p}_{v}^{j}-2{p}_{i}^{j+\frac{1}{2}}+{p}_{v}^{j+1}}{2+\beta }+\frac{1}{2}{p}_{0}.
\end{split}\end{equation*}

### Computational method

The angiogenesis and fluid models were implemented in MATLAB and computations were performed using a 64-bit Intel (R) Core (TM) processor (3.40 GHz) with 64 GB RAM. The computational domain was 6 × 6 × 6 mm^3^, which was discretized into a structured grid with uniform spacing of 150 μm along each orthogonal axis. Using this hardware and discretization parameters, each simulation of vascular network took approximately 90 s. The system of linear equation generated in the fluid flow model was solved in MATLAB to resolve vascular and vessel surface IFP. The tissue space was discretized into a structured grid with a uniform spacing of 85 μm where at each point the IFP was determined as described in Eq. 30.

### Model parameters

The parameters used to model the tumour geometry are given in Table 3. In the experimental work that Anderson and Chaplain based their angiogenesis model on, the average distance from the tumour to the parent vessel was 1–2 mm, hence a length scale of *L* = 1.5 mm is assumed in this study.

**Table 3:** Tumour geometry parameters

Parameter	Unit	Value	Reference
Length from tumour to parent vessel	mm	1.5	Stokes and Lauffenburger 1991
Length of domain *L*_D_	mm	6	Stokes and Lauffenburger 1991
Minimum vessel diameter *D_min_*	μm	10	McDougall *et al*. 2002
Parent vessel diameter *D_max_*	μm	30	McDougall *et al*. 2002
Tissue density	kg/m^3^	1050	Gas 2017; Jensen *et al*. 2008

Vessel hydraulic conductivity is based on the ability of the vessel to allow fluid movement across the vessel wall. Capillary filtration measurements in rat implanted tumours showed hydraulic conductivity values ten times higher than those reported in normal tissue. Vessels in the normal and tumour tissue region were prescribed vessel hydraulic conductivities. A summary of key parameters used in fluid flow simulations are given in Table 4.

**Table 4:** Fluid flow parameters

Parameter	Definition	Unit	Tumour	Normal	Reference
*L_p_*	Vessel hydraulic conductivity	m/Pa·s	$ 2.1\times {10}^{-11} $	$ 2.7\times {10}^{-12} $	Sevick and Jain 1991
*K*	Tissue hydraulic conductivity	m^2^/Pa·s	$ 3\times {10}^{-14} $	$ 3\times {10}^{-15} $	Baxter and Jain 1989
*π_v_*	Osmotic vascular pressure	mmHg	20	28	Stohrer *et al*. 2000
*π_i_*	Osmotic interstitial pressure	mmHg	15	8	Stohrer *et al*. 2000
*μ*	Blood viscosity	Pa·s	0.004	0.004	Nugent and Jain 1984
*p_arterial_*	Arterial pressure	mmHg	25	−	Jain 1988
*p_venous_*	Venous pressure	mmHg	10	−	Jain 1988
*p_0_*	Tumour surface pressure	mmHg	0	−	Chary and Jain 1989

### Morphological and hemodynamic analysis

In order to validate the tumour network generation model and the fluid flow model, a list of morphological and hemodynamic parameters were calculated and then compared with literature values obtained from empirical studies. For morphological analysis, the properties, tumor volume (*V*_t_), vascular density (*V*_d_), length density (*L*_D_), vessel surface to volume ratio (*S*/*V*), and maximum extravascular diffusion distance (*R*), were determined (Kim *et al*. 2012; Stamatelos *et al*. 2014):

31 $
\left\{{ \begin{aligned} &
{V_{\rm{t}}} = \dfrac{4}{3}\pi r_t^3,\\&
{V_{\rm{d}}} = \dfrac{1}{{4{V_{{\rm{tissue}}}}}}\displaystyle\sum \nolimits_{i,j}^N \pi  D_{ij}^2{L_{ij}},\\&
{L_{\rm{D}}} = \dfrac{1}{{{V_{{\rm{tissue}}}}}}\displaystyle\sum \nolimits_{i,j}^N {{L_{ij}}},\\&
S/V = 4\dfrac{{\displaystyle\sum \nolimits_{i,j}^N \pi  {D_{ij}}{L_{ij}}}}{{\displaystyle\sum \nolimits_{i,j}^N \pi  D_{ij}^2{L_{ij}}}},\\&
R  = \dfrac{1}{{\sqrt {\pi {L_{\rm{D}}}} }},
\end{aligned}}\right.

$

where $ {r}_{t} $ is the radius of the tumor tissue, $ {D}_{ij} $ and $ {L}_{ij} $ are the diameter and length of the vessel segment between nodes *i* and *j*. The hemodynamic parameters, mean velocity (*u_ij_*), mean shear stress (*τ_ij_*), vascular segment transit time (VSTT), and flow weighted mean path length (MPL), were calculated to validate the fluid flow model:

32 $
\left\{\begin{aligned} &

{{u_{ij}}}  = \dfrac{{4{Q_{ij}}}}{{\pi D_{ij}^2}},\\&
{{\tau _{ij}}}  = \dfrac{{32{\mu _{ij}}{Q_{ij}}}}{{\pi D_{ij}^3}},\\&
{{\rm{VSTT}}} = \dfrac{{\pi D_{ij}^2{L_{ij}}}}{{4{Q_{ij}}}},\\&
{{\rm{MPL}}}  = \dfrac{{\displaystyle\sum\nolimits_{i,j}^N {{Q_{ij}}} {L_{ij}}}}{{\displaystyle\sum\nolimits_{i,j}^N {{Q_{ij}}} }}.
\end{aligned}\right.

$

## Conflict of interest

Moath Alamer and Xiao Yun Xu declare that they have no conflict of interest.
